# 4D imaging of a porous carbonate rock under triaxial loading: quantitative correlation between local porosity and strain

**DOI:** 10.1107/S1600577526006958

**Published:** 2026-08-03

**Authors:** Catherine Doré-Ossipyan, Adriana Quacquarelli, Michel Bornert, Jean Sulem, Alexandre Dimanov, Patrick Aimedieu, Vincent De Greef, Andrew King

**Affiliations:** a Laboratoire Navier Ecole Nationale des Ponts et Chaussées 6–8 avenue Blaise Pascal 77420Champs-sur-Marne France; b Laboratoire de Mécanique des Solides, Institut Polytechnique de Paris, Route de Saclay, 91120Palaiseau, France; c Synchrotron SOLEIL CNRS, L’Orme des Merisiers, Départementale 128 91190Saint-Aubin France; University of Malaga, Spain

**Keywords:** microtomography, *in situ*, porous media, digital volume correlation, strain

## Abstract

The study of the mechanical behaviour of carbonate rocks has benefited from the evolution of experimental tools, due to their complex multi-scale microstructure. Triaxial cells transparent to X-rays have emerged and allowed *in situ* investigation of progressive failure developments and their link with microstructure. We conducted a series of 4D X-ray computed tomography mechanical tests on porous Saint-Maximin limestone at the PSICHE beamline of Synchrotron SOLEIL. Tests were performed using an in-house-developed triaxial press, Modulo. Volumes were scanned using a helical acquisition and reconstructed at 2.83 µm. They were then analysed using digital volume correlation. Volumetric strain and shear strain magnitude maps were computed using a 100 voxels gauge length with *Paraview*. Additionally, we computed porosity maps based on mean grey levels, at the same gauge length. Analysis of total and normalized incremental strain maps was carried out. We also compared volumetric strain maps with the initial porosity maps. Each step of the procedure is described herein. The errors on strain and porosity are discussed.

## Abstract 2

A 4D X-ray computed tomography experimental campaign was carried out on a porous carbonate rock to investigate the link between initial porosity and deformation modes. This paper presents the experiment and the methods used to explore this relationship.

## 1. Introduction

Micromechanisms involved in deformation processes of porous geomaterials have been widely studied for several decades by using *in situ* or *post mortem*X-ray imaging and full field methods (Abdallah *et al.*, 2021 ▸; Ando, 2015 ▸; Bésuelle *et al.*, 2000 ▸; Chen *et al.*, 2020 ▸; David *et al.*, 2007 ▸; Desrues & Viggiani, 2004 ▸; Flavigny *et al.*, 1990 ▸; Huang *et al.*, 2019 ▸; Kandula *et al.*, 2022 ▸). Experimental tools have become more sophisticated, providing a better description of continuous strain development, and linking it with microstructure, micromechanisms and deformation modes. The study of mechanical processes in carbonate rocks greatly benefits from this progress. The complex geological processes involved in limestone formation result in a wide variety of microstructures, even within the same rock. This makes synchrotron 4D X-ray computed tomography experiments a precious tool for failure investigation in porous carbonate rocks. But the decreasing difficulty in obtaining spatially resolved images increases the demands of data analysis, leading to challenges of large data-set storage and visualization. Such problems are typically encountered with ‘big data’.

We led an *in situ* experimental campaign at the PSICHE beamline (Synchrotron SOLEIL, Palaiseau) on 12 samples of Saint-Maximin limestone (Doré-Ossipyan *et al.*, 2025 ▸), producing 80 TB of data. Saint-Maximin limestone samples of diameter 8 mm were tested under axisymmetric conditions with a recently developed mini triaxial press, called Modulo. Volumes were acquired at 2.83 µm, using a helical scanning procedure. Analysing the entire data set is impractical and resource intensive.

In this paper, we propose an investigation method used to study the relationship between local porosity and volumetric deformation. The reconstructed images were first analysed by digital volume correlation (DVC) (Bay *et al.*, 1999 ▸; Bornert *et al.*, 2004 ▸) to obtain three-dimensional local strain maps at the 100 voxels scale. We computed incremental and total volumetric strain and shear strain magnitude maps. We present here the DVC post-processing that we developed to visually describe the obtained strain maps. Instead of image segmentation, we used grey level as a pr­oxy for porosity. The correlation domain positions are used to calculate the mean grey level over the same 100 voxels gauge length. We characterized local micromechanisms and porosity at a scale of 283 µm at any point in the sample for each loading step. The errors on porosity, strain and correlation between initial porosity and volumetric strain are quantified. This method ensures that any correlation would likely be a consequence of an intrinsic property rather than randomness. The method described in this paper can be used in its current form or adapted for any other material.

## 2. Experimental methods

### 2.1. Sample selection with preliminary X-ray computed tomography

**Figure 1:**
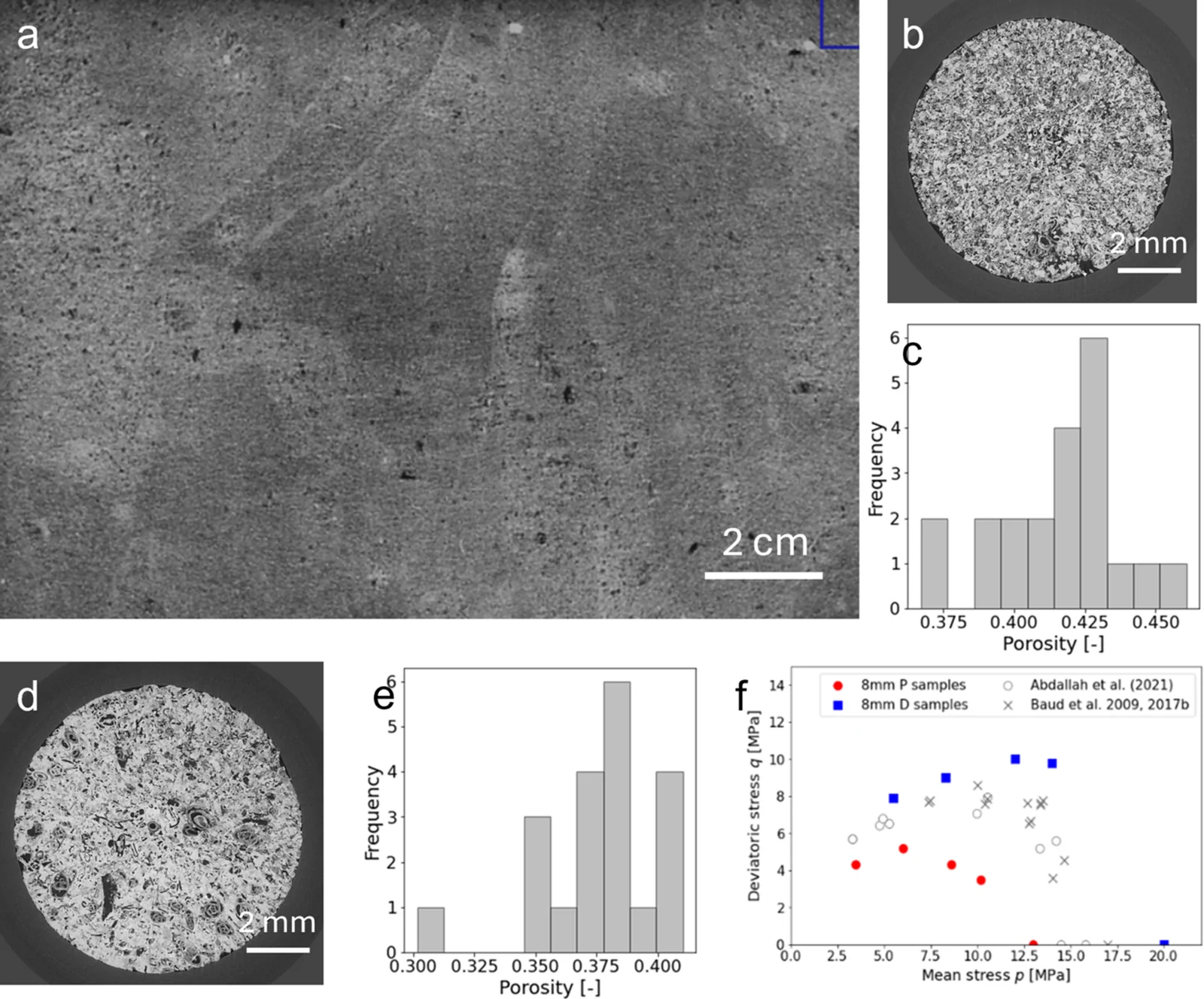
(*a*) X-ray computed tomography image of a 16 cm × 30 cm parallelepipedal SML block used to core 8 mm samples. The image was acquired at 16 µm. (*b*, *d*) Vertical cross sections of an 8 mm-diameter porous sample and a dense sample, with a 2.83 µm voxel size. (*c*, *e*) Initial global porosity distribution of porous and dense samples. (*f*) Critical yield points of the 8 mm samples (blue squares for dense ones and plain red circles for the porous samples). The results are compared with 40 mm-diameter samples (grey circles and crosses).

The studied material was Saint-Maximin limestone (SML), a high porosity (38%) biocalcarenite. Microstructural heterogeneity at the centimetre scale was observed in the provided block, with alternating high and low porosity zones (Abdallah *et al.*, 2021 ▸). These can be differentiated based on their grey levels in an X-ray computed tomography (XRCT) image [Fig. 1 ▸(*a*)]: dense zones appear brighter than porous ones. We aimed to characterize the mechanical behaviour of the dense and porous zones individually.

A parallelepipedal SML block of 16 cm × 30 cm provided by the Rocamat quarry in Saint-Maximin-sur-Oise, North of Paris, was imaged at 16 µm with an UltraTom microtomograph (RX Solutions, Chavanod, France) at the Navier Laboratory. The block is the same as that used in the work of Abdallah *et al.* (2020 ▸, 2021 ▸). Zones, where cylindrical samples of 8 mm × 16 mm could be cored, were identified. The obtained porous and dense samples had distinctive microstructures [Figs. 1 ▸(*b*) and 1(*d*), respectively]: larger grains and more cementation in dense zones, and abundant intergranular porosity and smaller grains in porous zones. On average, dense samples had indeed lower porosities than the porous ones [Figs. 1 ▸(*c*) and 1(*e*)]. But the porosity difference is relatively small: the terms dense and porous are not related to an absolute threshold, but to the different microstructures. Finally, mechanical tests showed that the dense samples consistently had a higher elastic limit than the porous ones, showing a first-order control of initial porosity [Fig. 1 ▸(*f*)].

### 2.2. Modulo: a modulable triaxial testing device for *in situ* testing

Mechanical tests [Fig. 1 ▸(*f*)] have been performed using a small triaxial press specially designed for 4D investigations of deformation modes in geomaterials. Recent advancements in X-ray-transparent triaxial deformation apparatuses have enabled significant progress in studying strain localization mechanisms and coupled thermo-hydro-mechanical processes in rocks. High-flux and high-energy synchrotron beamlines, such as those at the Advanced Photon Source or ESRF, allow titanium-made presses, covering broader stress conditions than aluminium-made ones. Our triaxial apparatus was developed at the Laboratoire de Mécanique des Solides (Ecole Polytechnique, France) to address different scales involved in deformation and damage under triaxial loading and in dry or wet conditions.

Modulo, the miniature *in situ* triaxial press, was designed to conduct experiments in a laboratory microtomograph or at a synchrotron. The triaxial apparatus is identical to a conventional triaxial press, except for its significantly smaller size and transparency to X-ray confining cells. Such apparatuses are already present in other laboratories, with various characteristics: the press developed at 3SR, Université Grenoble Alpes, France (Viggiani *et al.*, 2004 ▸), the HADES rig (Renard *et al.*, 2016 ▸), the mini triaxial HP/HT press presented by Voltolini *et al.* (2019 ▸), a low-cost press (Fusseis *et al.*, 2014 ▸), the Mjölnir press (Butler *et al.*, 2020 ▸) and Heitt Mjölnir (based on the latter) (Freitas *et al.*, 2024 ▸).

**Figure 2:**
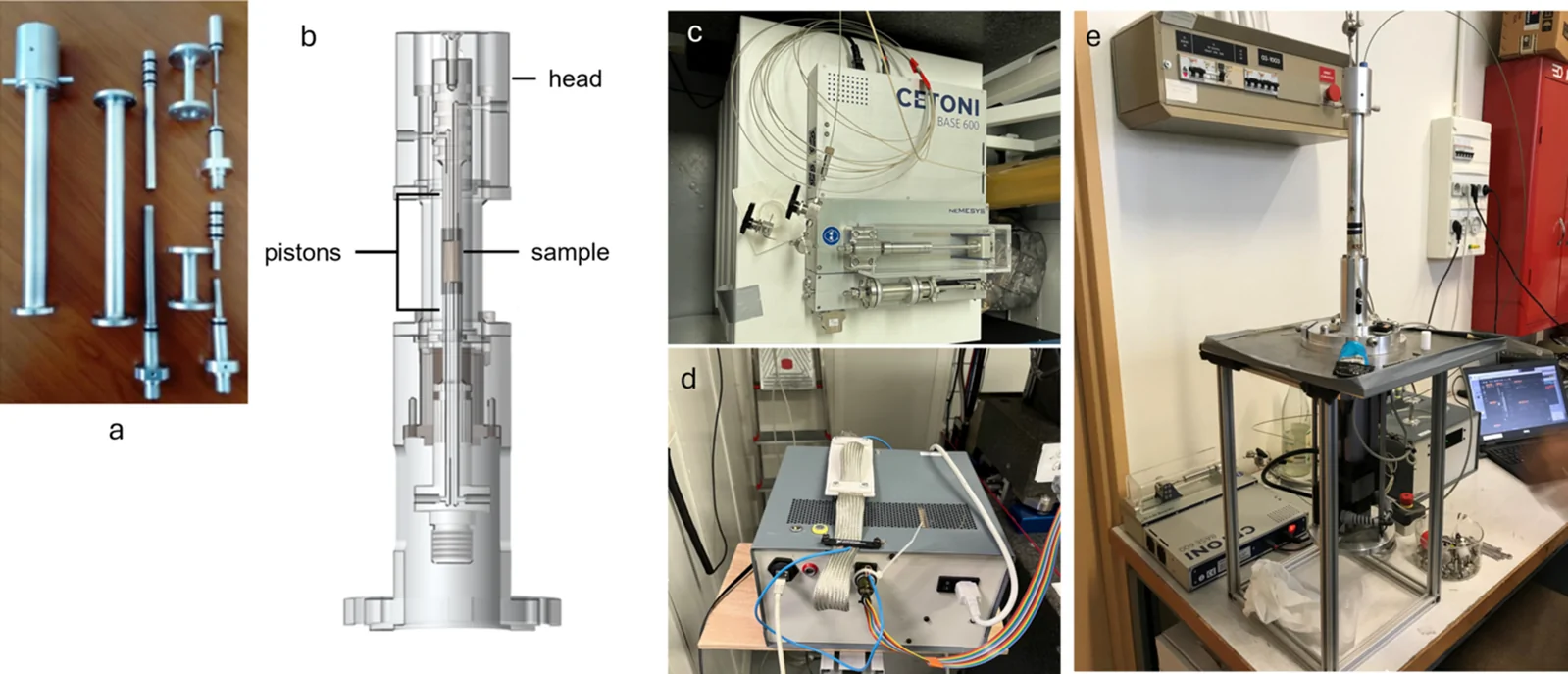
(*a*) Different available sizes for the pistons and cells (non-exhaustive) for the triaxial press. (*b*) Cross section of the triaxial press body set for 8 mm samples. (*c*) CETONI injection 3 ml pump. (*d*) Controller. (*e*) Triaxial press set for 15 mm-diameter samples, with the CETONI injection pump and the controller behind the press.

The present triaxial loading device is composed of a uniaxial electromechanical actuator equipped with a force sensor, different confining pressure cells and pistons, a water injection pump, and a pressure sensor. The geometry of the press is designed to minimize the source–object distance and achieve the best spatial resolutions in a laboratory micro-computed tomography (fan geometry of the beam), less critical under synchrotron radiation (parallel geometry) (Lenoir *et al.*, 2007 ▸). The originality of this press lies in the modularity of its components, which can be adapted to meet the requirements of individual *in situ* experiments. Different piston sizes are available: 4, 8, 12 and 15 mm in diameter [Fig. 2 ▸(*a*)]. Chamber cells for each piston size, adapted for laboratory microtomographs and synchrotron imaging, are also available. In addition to its size modularity, the system is designed to allow independent interporal fluid control.

The press is made of aluminium. The walls have a thickness of 2 mm, allowing a maximum 20 MPa of confining pressure at ambient temperature. Under the body is a ball screw linear actuator, driven by a stepper motor. The motor activates a bottom piston, which applies axial pressure and is controlled in micro-steps. Displacement is calculated from the micro steps, giving a qualitative sample deformation. The piston is monitored via two force sensors. The uniaxial rig is managed by *LabVIEW* software, where control is force- or displacement-rate driven.

The sample is slipped into a rubber jacket and placed between the two aluminium pistons. The top piston is inserted into a head [Fig. 2 ▸(*b*)], attached to the confining cell. A pressure sensor with a 0.05 MPa precision and a PEEK tube are attached to this head. The other end of the PEEK tube, with a 0.75 mm internal diameter, is connected to the water injection pump. The pump is a stainless steel Nemesys High Pressure syringe pump (CETONI) with a 3 ml capacity [Fig. 2 ▸(*c*)]. The pump is managed via *Qmix* software. The flow rate was controlled manually during the experiment, but *Qmix* allows user programs for specific needs. During hydro­static loadings, the flow rate was progressively decreased with increasing confining pressure. The uniaxial rig is independent of the pump and supports a maximum load of 10 kN. For hydro­static loadings, the axial force can be servo-ed to the pressure sensor mounted on top. Before a triaxial loading, the piston is freed from the confining pressure.

The motor command and the measurement acquisition are ensured by a controller from the National Instruments compact RIO series [Fig. 2 ▸(*d*)], connected via Ethernet to a PC with two software packages: the first one retrieves the measurements, and allows the user to save and display them; the second one displays the user interface of the code running on the cRIO controller. The 15 mm sample diameter setup is shown in Fig. 2 ▸(*e*).

### 2.3. Experimental setup at PSICHE

**Figure 3:**
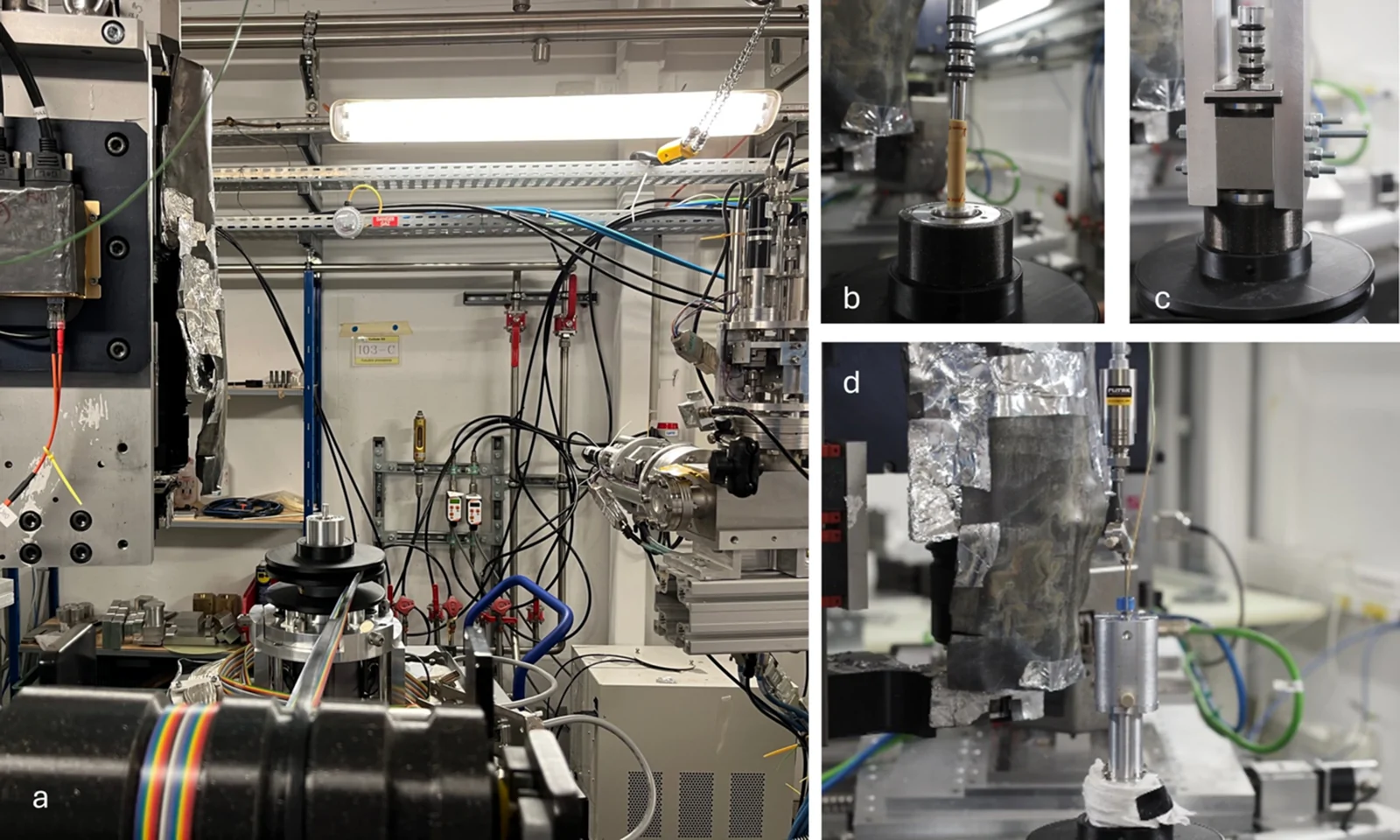
(*a*) Triaxial press base set in the optical hutch of the PSICHE beamline (Synchrotron SOLEIL) on the X-ray beam path, with the detector on the left. (*b*) 8 mm sample in its jacket, with copper wires tightening the silicone jacket against the pistons. (*c*) Mounting aid allowing to carefully screw the top piston inside the head and control the force applied on the sample. (*d*) Final setup, with the PEEK tube and the confining pressure sensor attached to the head.

The press with the 8 mm setup was placed inside the synchrotron optical hutch of the PSICHE beamline [Fig. 3 ▸(*a*)] on the tomograph’s rotation table (King *et al.*, 2016 ▸). The 8 mm samples were placed between pistons inside the cell, isolated by a rubber jacket, and copper wires were used to tighten the membrane against the pistons [Fig. 3 ▸(*b*)]. Polished aluminium stubs were placed between the sample and the pistons. A special device for sample placement was also developed to avoid damaging the sample surfaces during the sample mounting [Fig. 3 ▸(*c*)]. The mounted setup is shown in Fig. 3 ▸(*d*).

**Figure 4:**
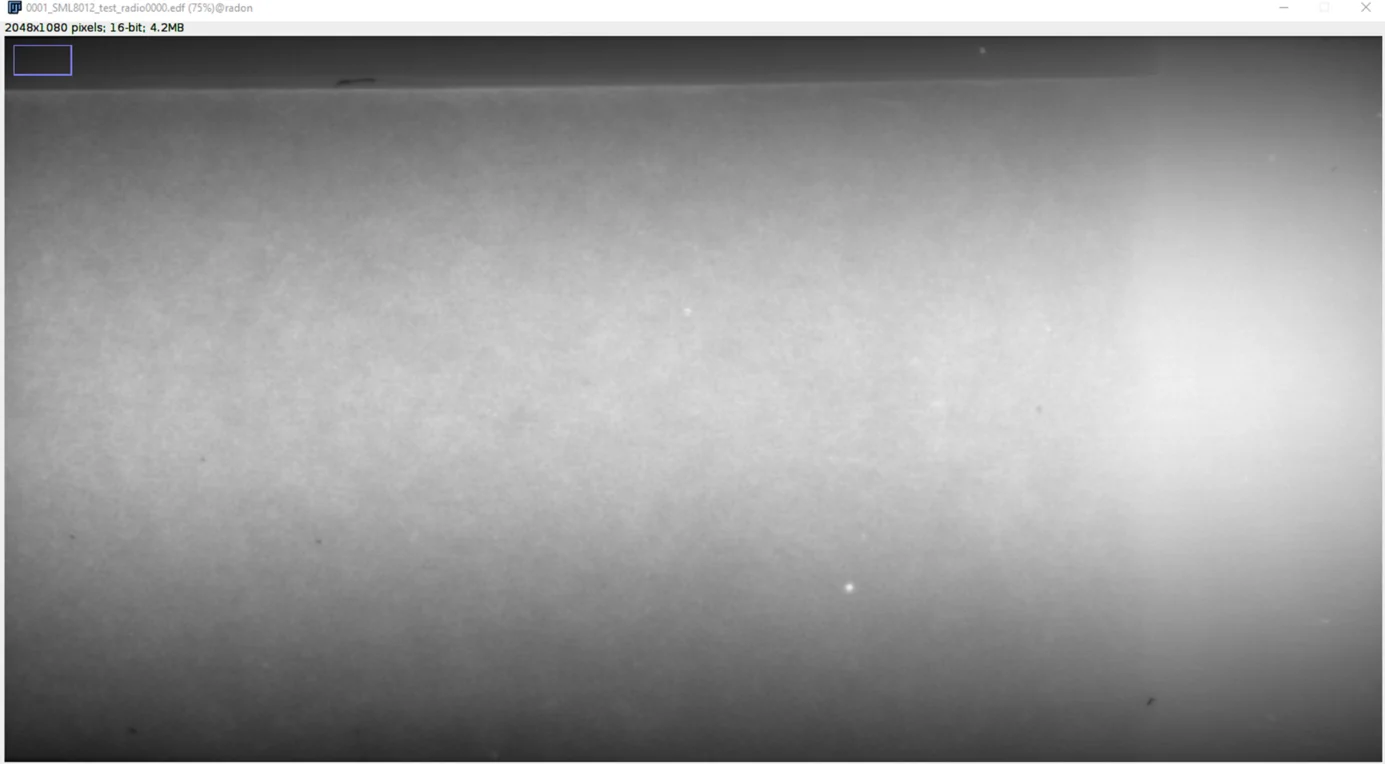
Half 2048 × 1080 projection of an 8 mm sample imaged on the PSICHE beamline at Synchrotron SOLEIL.

The PSICHE beamline is equipped with a tomography instrument, which consists of X-ray optics, mechanical stages, a detector system, instrument control and data analysis systems. The selected operating mode of the beamline is the pink beam mode, which provides high flux tomography. In our configuration, the beam size is around 3 mm vertically, which allows scanning samples of 18 mm maximum. The tomograph detector uses a sensor coupled to a 250 µm LuAG:Ce scintillator. A Hamamatsu Orca Flash4.0 V3 camera serves as the CCD sensor and has a region of interest of 2048 × 1080 pixels (Fig. 4 ▸).

Beam attenuation was performed by a 0.7 mm tungsten filter. For the helical scan requirements, the press needed to sustain eight rotations around itself per scan, multiplied by a total of 200 loading steps. The motor was connected to the controller [Fig. 2 ▸(*d*)] via an 8 m-long ribbon cable. A braided cable sleeve was added on the connectors to avoid interferences between the signals and the X-rays. A homemade support system was also constructed, consisting of a stand with 3D printed pulleys, weights and reels for the flat cables. This allowed for a tight winding and unwinding during helical scans and prevented rapid wearing of cables. Another method has also been reported: a system of slip rings and embarked pumps was proposed for the HADES rig at ESRF on ID19 and BM18 (Renard *et al.*, 2016 ▸).

### 2.4. Helical acquisition

Helical computed tomography (HCT) was used to continuously scan the whole sample in about 12 min, despite the small height (3 mm) of the X-ray pink beam. In pink beam mode, the exposure time is 7 ms per projection, with a mean energy of 66 keV. Helical acquisition was completed with eight turns of 360° for a total height of 21.6 mm, with 4200 projections per turn. For practical reasons, each scan was began at the previous final position: volumes would either be acquired clockwise or counterclockwise.

The voxel size of 2.83 µm ensures a detailed view of the micromechanisms acting at grain scale (∼150 µm on average). The vignetting observed in Fig. 4 ▸ is certainly caused by the beam profile. This causes virtual local density variations, and can be problematic for quantitative use of grey levels. A flat-field correction performed on an aluminium plate or the empty triaxial press, instead of a standard flat field (empty beam trajectory), could have probably reduced such effects. Images were reconstructed with *PyHST2* (Mirone *et al.*, 2014 ▸) and analysed without any ring artefact correction.

## 3. Local porosity maps

At 2.83 µm, the high content of a microporous phase (aggregate calcite grains and sparritic cement) bridges the grey level (GL) peaks associated with dense calcite (lowest GL peak) and open pores (highest GL peak). Instead of image segmentation (Ji *et al.*, 2012 ▸, 2015 ▸), we chose to compute porosity maps based on the mean GL method presented in the work of Y. Abdallah (Abdallah, 2020 ▸; Abdallah *et al.*, 2021 ▸). Since the number of samples and steps have largely increased, the method is simplified to reduce data redundancy. Porosity maps are computed as a file containing positions and porosity, resulting in a compressed version of the original volume. We suggest adding the local mean GL and the local standard deviation calculation during the image correlation procedure.

### 3.1. Procedure

Ideally, the grey level *g*_v_ of a voxel is an affine function of the grey levels of each individual phase. When averaging over the whole volume, we obtain
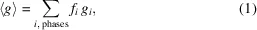
where 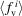 = 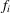 is the overall volume fraction of phase *i*. In the case of SML, it is made of three chemical phases: void (pores), calcite and quartz. However, at 66 keV and a 2.83 µm voxel size, calcite and quartz (representing approximately 10%) can be considered to have similar transmittance coefficients. They are treated as a unique solid phase. Consequently, we obtain the following relation, where φ is the global porosity,
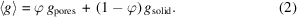
Finally, in a two-phased porous material, variations in local porosity result in local density variation. If the imaging conditions remain the same between two states, the evolution of the mean grey level reflects the porosity evolution.

The relationship in equation (2) is valid not only on the global scale but also on a local scale. Knowing the global porosity φ and its mean grey level 〈*g*〉, the mean grey level *g*_pore_ of a pore, we deduce from it the mean grey level *g*_solid_ of the solid phase,
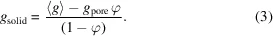
The grey value of each subdomain *i* of size *V* is then used to deduce the porosity,
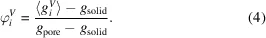
We computed local porosity maps by binning volumes by 100 times in each direction (coinciding with the DVC positions and correlation domain size): the original image has a size of 88.88 Gb, and the compressed image is only 91 kb.

### 3.2. Multi-scale porosity characterization

**Figure 5:**
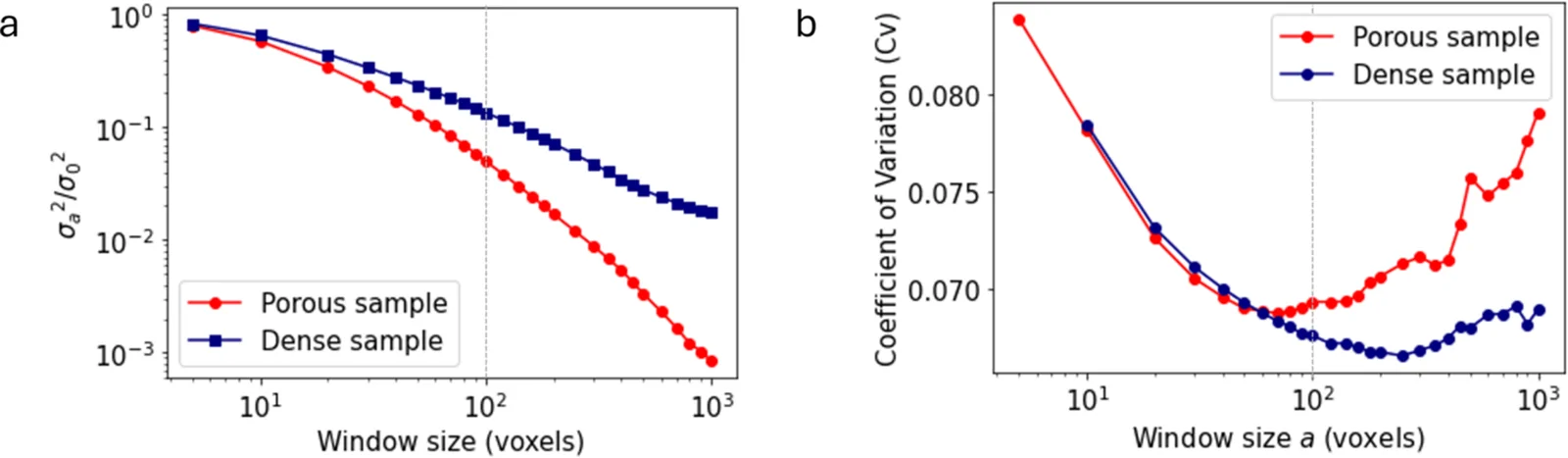
(*a*) Variance attenuation induced by binning, quantified by the ratio 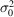, where 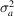 and 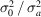 are the original and filtered variance, respectively. (*b*) Mean coefficient of variation computed for binned fields filtered with local mean and standard deviation. This coefficient reflects persistence of structures larger than the window size. A minimum is observed when the dominant scale heterogeneities become homogenized.

We suppose an image has a grey level variance 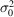. We apply a local mean grey level filter computed over a window of size *a*, and compute the resulting variance 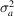. The ratio 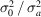 indicates how much of the original heterogeneity remains after filtering. Values close to 1 indicate that structures responsible for porosity fluctuation are larger than *a*. Lower values indicate that filtering strongly reduces the fluctuations. For dense and porous microstructures [Fig. 5 ▸(*a*)], the ratio decreases monotonically with increasing window size. For a window size of 100 voxels, approximately 95% of the initial variance of the porous sample is reduced, and 90% for the dense sample.

To further characterize the spatial organization of porosity, we analyse the scale-dependent coefficient of variation computed over filtered fields. In Fig. 5 ▸(*b*) we plot the mean ratio between the binned image filtered with a local mean and the same one binned with a local standard variation filter, computed for the same window size *a*, for the porous and dense samples. Both microstructures show a non-monotonic evolution with the window size, reflecting competing scales. The evolution is characterized by an initial decrease, followed by a minimum and a steeper increase for the porous sample than for the dense one. The minimum is evaluated at 60–80 voxels for the porous sample, followed by a strong increase, indicative of multi-scale organization of porosity. The minimum is evaluated at 200–300 voxels for the dense sample. This suggests a larger characteristic correlation length. The choice of 100 voxels appears to lie in an intermediate homogenization regime for both materials.

### 3.3. Effect of the beam profile on local porosity values

A worst-case local porosity standard deviation can be estimated by performing an image subtraction between two images of an undeformed sample scanned clockwise or counterclockwise (see explanation given in Section 4.3). Image subtraction (Chateau *et al.*, 2018 ▸; Chen *et al.*, 2021 ▸) is typically used to reflect local evolutions of microstructure at the voxel scale and is based on DVC. Here, the GL variation would directly reflect the local porosity reduction between two states.

**Figure 6:**
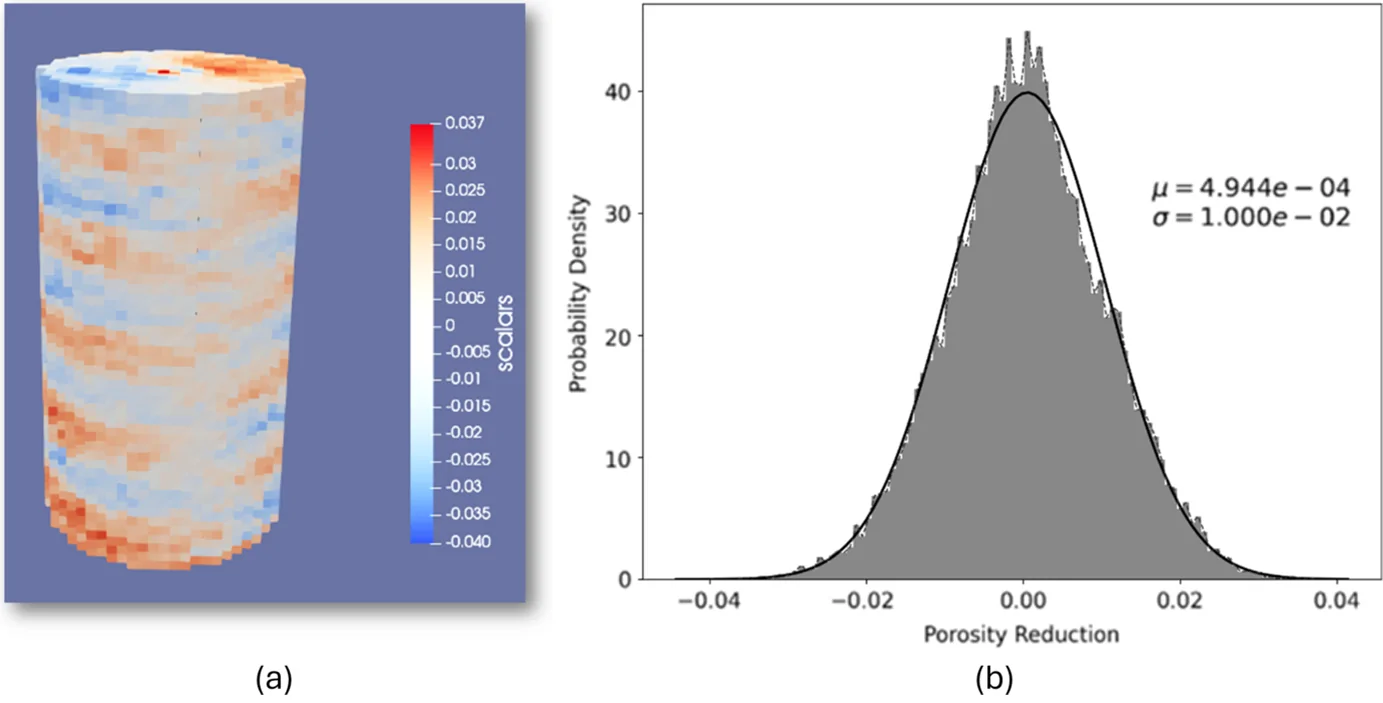
Typical local porosity subtraction. (*a*) Local porosity reduction map at a scale of 100 voxels; and (*b*) the corresponding probability density with a fitted normal distribution.

Fig. 6 ▸ presents a typical local porosity reduction map (*a*) and a histogram of probability densities (*b*). An alternating regular pattern of denser and more porous zones is visible, revealing a systematic error. The pattern period is consistent with the detector size. In addition, the maximum local porosity reduction is of the order of 4% in absolute value. The local porosity reduction density follows a normal distribution centred over μ = 4.9 × 10^−4^ with a standard deviation of σ = 1 × 10^−2^. 90% of the local porosity evolution errors range between −2 × 10^−2^ and 2 × 10^−2^. The calculated errors are important and only the initial local porosity map was used as the quantitative microstructural parameter. In case of a quantitative use of local porosity maps, these patterns can be removed by subtracting the porosity reduction maps from the porosity maps. To remove the pattern pixel-wise, a DVC must be performed first [see Chateau *et al.* (2018 ▸) and Chen *et al.* (2018 ▸) for a detailed process].

## 4. Digital volume correlation

### 4.1. Principle

DVC (Bay *et al.*, 1999 ▸; Bornert *et al.*, 2004 ▸; Gaye, 2015 ▸; Lenoir *et al.*, 2007 ▸; Zinsmeister, 2014 ▸) is a three-dimensional extension of digital image correlation (DIC) (Doumalin, 2000 ▸; Sutton *et al.*, 1983 ▸). This method consists of determining the displacement field from a comparison of two three-dimensional digital images, before and after deformation, or between two different deformed states. Subdomains, or correlation domains, are defined in the reference image and are each identifiable by their grey level distribution (GLD). We look for the best transformation that associates the closest GLD of voxels (three-dimensional pixels) in the deformed state to the reference GLD. This assumes that we have sufficiently contrasted images at the studied scale and that each area has a unique GLD in a close neighbourhood. Heterogeneous materials such as porous carbonates are very suitable for the use of DVC.

The similarity between two configurations is evaluated using a correlation coefficient. Let *f* and *g* be the GLD in the reference and deformed images, respectively. A correlation coefficient *C*(ϕ_*i*_) of the material transformation ϕ_*i*_ is calculated between the domains 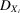 and 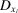, which is given by the formula (Doumalin & Bornert, 2000 ▸)
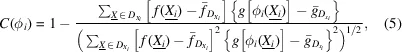
where 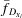 and 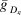 are the averages of the grey levels over 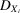 and 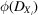. 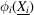 has integer values with respect to the voxels. Subvoxel precision is obtained by interpolating GL of the closest voxels.

### 4.2. Procedure

**Figure 7:**
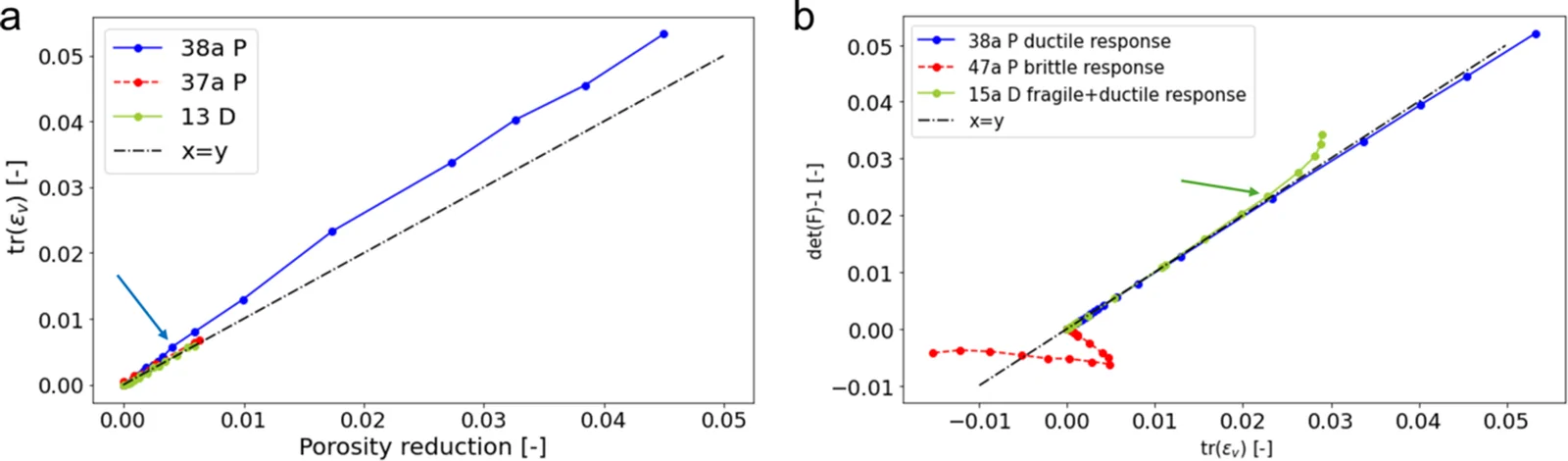
(*a*) Porosity reduction, obtained by subtracting mean grey levels, versus volumetric strain for three samples. (*b*) 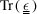 versus det(*F*) − 1 for three samples failing in different regimes. The arrows in both graphs indicate onset of plasticity, correlated with a deviation from linearity, illustrated by the *x* = *y* line. P = porous sample, D = dense sample.

Here we describe the practical procedure followed to obtain 3D strain maps. The displacement maps are obtained using an in-house-developed software, *CMV3D*, written in C language (Bornert *et al.*, 2004 ▸; Lenoir *et al.*, 2007 ▸), and are post-processed in *Paraview*. *CMV3D* runs on 8-bit images, and volumes are converted from 16-bit format. We can reasonably consider that the contrast and brightness parameters were stable during a mechanical test: the trends between global volumetric strain and average grey level (average porosity) for each sample systematically showed a linear trend, with a slope of 1, during the elastic response of the material [Fig. 7 ▸(*a*)]. The deviation observed for the sample 38a (blue arrow) corresponds to the onset of plasticity and could be attributed to the presence of quartz. The deviation is of the order of 10^−3^ and remains acceptable. Each final image has a size of between 60 and 100 Gb.

A regular mesh is defined in the reference state. The mesh forms a parallelepiped which envelops the cylindrical sample, and it is defined by manually selecting four vertices. Only positions inside the cylinder are analysed. This leads to a cylindrical region of interest, typically containing 40000 cubic correlation domains of 100 voxels (283 µm). The analogous positions are then determined in the next deformed image, with a 0.4 correlation threshold. This step is performed semi-automatically. The approximate position of a point in the deformed image is searched around the nearest known point by optimizing the correlation coefficient *C*(φ) for all integer positions around a search domain of size 5 × 5 × 5 voxels. In case of non-convergency (strain too large, strong microstructural changes, or small contrast) the point is skipped. The third step, the subvoxel optimization, is done automatically, and can be combined with the previous step. The non-converged points are then interpolated using the nearest converged points.

A regular finite-element mesh is constructed, where nodes correspond to initial positions in the reference image. The nine components of the average deformation gradient are computed using finite-elements method. In the small strain framework, the trace of the strain tensor trace gives the volumetric strain 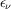 = 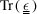. The shear strain intensity is calculated using the Von Mises equivalent strain γ = (2*e*_*ij*_*e*_*ij*_)^1/2^ where *e* is the deviatoric strain. All these steps are repeated for each increment. Volumetric strain can also be calculated using det(*F*) − 1, where *F* is the deformation gradient. In Fig. 7 ▸(*b*), we show a comparison between 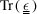 and det(*F*) − 1 for three samples showing different mechanicals responses: ductility (porous sample 38a in blue), brittleness (porous sample 47a in red) and a transitional behaviour characterized by both a fragile and ductile response (dense sample 15a in green). While 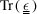 and det(*F*) − 1 show little to no difference in sample 38a presenting a ductile response, brittle behaviour induces a remarkable difference, invalidating the infinitesimal strain theory. The deviation is also observed for the sample 15a, during strain localization (green arrow).

For better efficiency, we calculate strains between two consecutive steps. This allows to better track microstructural changes that could be too large for DVC routines. However, some drawbacks stem from this:

(i) The first point in the second step of the DVC routine is to be indicated manually, as it would take too much time for a computer to find the first deformed position. As a result, each step must be launched manually, significantly increasing the length of the procedure.

(ii) A local torsion was observed in local strain maps of two consecutive scans. This point is discussed in the section below.

(iii) This can create error propagation. This error can be estimated by comparing a total strain map obtained from incremental steps with a second DVC calculation between the undeformed state and the considered deformed step.

DVC was performed on a computer with a 755 GB memory and a dual-socket server with two Intel Xeon Gold 6248R CPUs, totalling 96 threads. The subvoxel optimization is parallelized in *CMV3D*. One stage is calculated in approximately 5 h for 40000 correlation domains, but necessitates human intervention between previously described steps, so the full procedure can easily exceed a day with eventual setbacks. With the available memory, two calculations can be done at the same time, at the expense of CPUs. Scientific imaging would surely benefit from a further task automatization and advanced data compression algorithms.

### 4.3. Strain field error estimation

The error magnitude in a strain field or in local porosity maps are caused by many factors (XRCT noise, reconstruction artefacts, correlation domain size, grey level interpolation, displacement field discretization, *etc*.). They can be evaluated by performing a comparison of two undeformed or identical states. Ideally, a zero-deformation tensor and no porosity evolution should be obtained locally and globally (see Section 3.2 for local porosity errors).

During the experiment, volumes were acquired either from bottom to top or top to bottom: clockwise or counterclockwise. We are presented with two cases: two states scanned in opposite travelling directions (odd configuration) or collinearly (even configuration).

#### 4.3.1. Odd configuration

**Figure 8:**
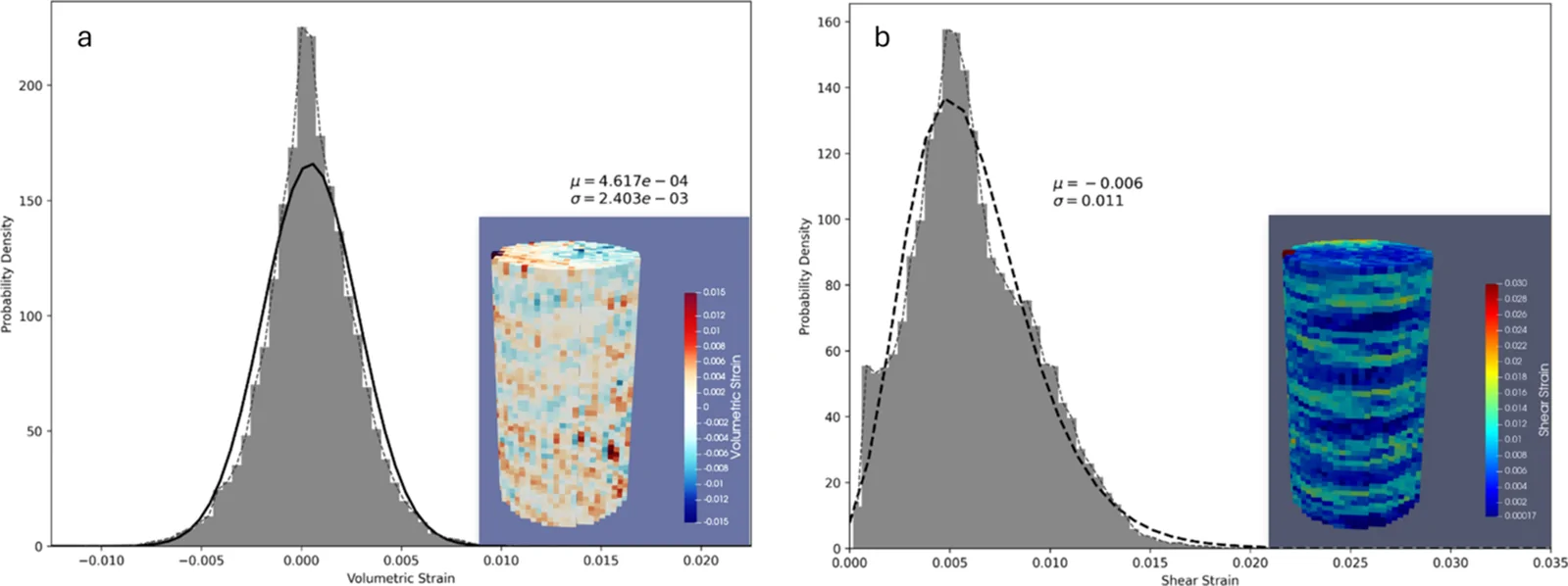
Typical DVC applied on odd images. (*a*) Probability density of volumetric strain with a fitted normal distribution, and the corresponding volumetric strain map. (*b*) Probability density of shear strain with a fitted lognormal distribution, and the corresponding shear strain magnitude map.

DVC is applied on reconstructed images of two extremely close consecutive steps taken in the elastic part of the hydro­static loading curve for an 8 mm sample tested under hydro­static conditions. The global strain tensor is evaluated at
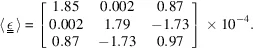
In Fig. 8 ▸ we show the probability densities of local volumetric (*a*) and shear strains (*b*). Volumetric strain follows a normal distribution centred around μ = 4.6 × 10^−4^ with a standard deviation of σ = 2.4 × 10^−3^. In addition, 90% of the volumetric strain errors range between −3.5 × 10^−3^ and 4.4 × 10^−3^. Regarding the shear strain magnitude, its logarithm follows a normal distribution of mean value μ = 6 × 10^−3^ and a standard deviation σ = 1.1 × 10^−2^. 90% of shear strain magnitudes are lower than 1.5 × 10^−2^. Locally, volumetric strain is at most of the order of 1.5% in absolute value [Fig. 8 ▸(*a*)], and shear strain can reach up to 3% [Fig. 8 ▸(*b*)]. Both volumetric and shear strain maps show a distinct pattern of local variations, revealing a systematic error, but they do not coincide (both maps have the same orientation).

#### 4.3.2. Even configuration

**Figure 9:**
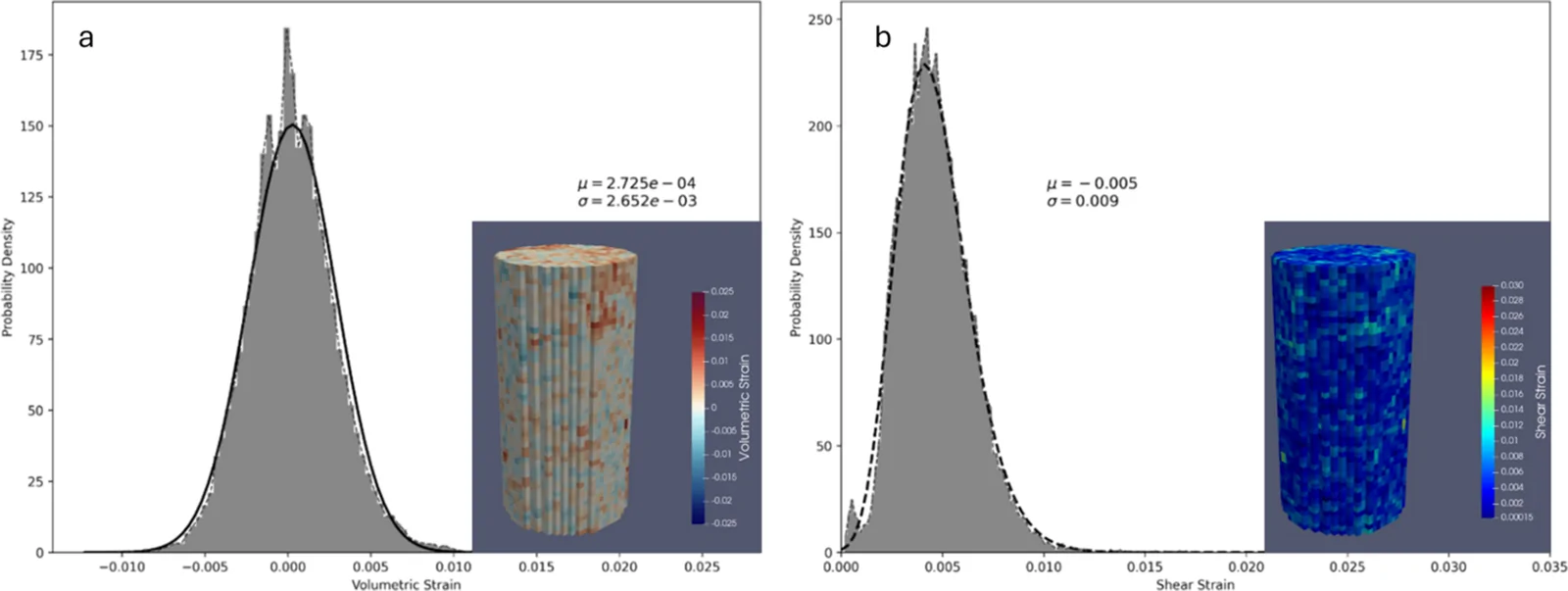
Typical DVC applied on even images. (*a*) Probability density of volumetric strain with a fitted normal distribution, and the corresponding volumetric strain map. (*b*) Probability density of shear strain with a fitted lognormal distribution, and the corresponding shear strain magnitude map.

A sample was loaded in hydrostatic conditions, and a cycle of unloading/loading was performed at a given pressure. We apply DVC on the two volumes representing the loaded state, acquired collinearly. The global strain tensor is evaluated at
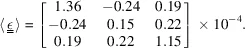
The local volumetric strain distribution follows a normal distribution centred around μ = 2.7 × 10^−4^ with a standard deviation of σ = 2.4 × 10^−3^. In addition, 90% of the volumetric strain errors range between −4.2 × 10^−3^ and 4.2 × 10^−3^. Regarding the shear strain magnitude distribution, its logarithm follows a normal distribution of mean value μ = 5 × 10^−3^ and a standard deviation σ = 9 × 10^−3^. 90% of shear strain magnitudes are lower than 1 × 10^−2^. Locally, volumetric and shear strain can both reach 1.5% [Figs. 9 ▸(*a*) and 9(*b*)]. No pattern is visible and the error is quasi randomly distributed, with local volumetric strain extrema correlated to maximum shear strain.

#### 4.3.3. Discussion

A systematic error is observed in the odd configuration but not in volumes acquired in the same direction (collinear). This surprising result might be caused by reconstruction errors that remain to be determined (*e.g.* vertical velocity variation of the sample stage in one direction or another, encoding errors of the projection angles).

At a ∼300 µm gauge length, the error order of magnitude on the local volumetric strain is 10^−3^, even with the systematic error in the odd configuration. Local shear strain magnitude error is, however, of the order of 10^−2^, which is significant. At the global scale (8 mm width × 16 mm height sample), the accuracy is one order better for both volumetric and shear strain. In the work of Abdallah (2020 ▸), the local deformation, calculated at a 40 voxels gauge length (∼1 mm) is of the order of 10^−3^. For global strain, determined over a cylindrical domain of 4 cm diameter times 4 cm height, the accuracy is one order better. Volumetric strain appears to be a more robust strain invariant than shear strain magnitude or GL-based porosity. Volumetric strain uncertainty decreases with higher spatial resolution, but shear strain error magnitude increases by one order (2.83 µm versus 24 µm). The choice of a better spatial resolution and lower scan time comes at the expense of ring artefacts and signal-to-noise ratio. The choice of the selected gauge length (100 voxels) constitutes an appropriate compromise between spatial resolution, measurement robustness and computational costs.

### 4.4. Material point description at a scale of 283 µm

Total strain maps were obtained without additional numerical computations. Incremental strain maps were post-processed to better visualize strain evolution without any mesh distortion. Incremental strains were projected back into their initial undisturbed configuration. This process is explained below.

#### 4.4.1. Local full strain tensor

**Figure 10:**
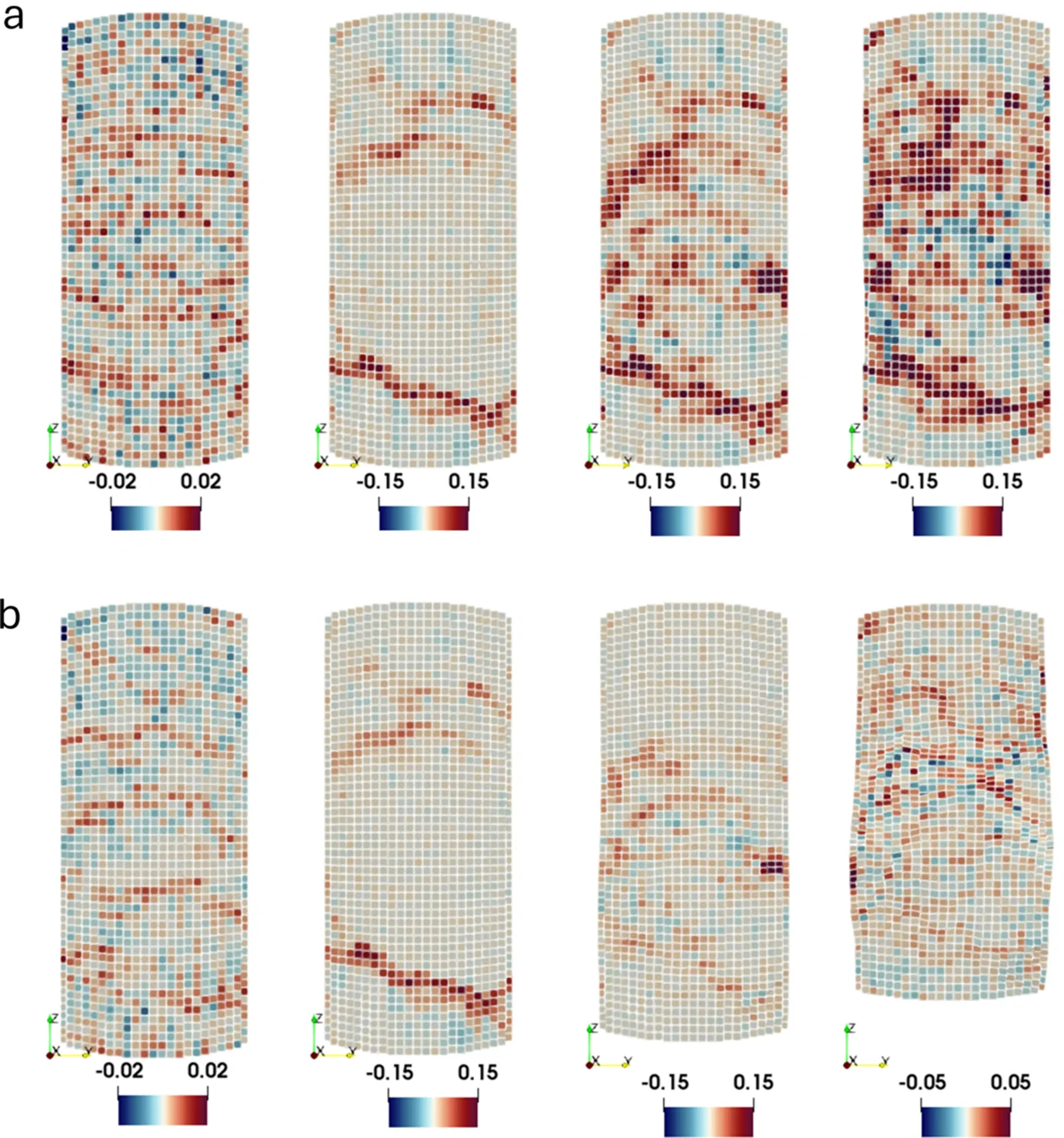
Successive total (upper row) and incremental (lower row) local volumetric strain maps, computed at 100 voxels gauge length, for a dense 8 mm sample tested at 6 MPa. Each column shows the same loading step. Blue represents dilation and red contraction.

DVC calculates the displacement field of material points, *i.e.* subvolumes or correlation domains, by minimizing a correlation coefficient [equation (5)]. We systematically applied DVC on two successive images, between *t* = *n* − 1 and *t* = *n*. Positions obtained for *t* = *n* are used to do the next DVC step, *i.e.* between *t* = *n* and *t* = *n* + 1. Such maps are referred to as *incremental* maps and illustrate local strain intensity between two deformed stages. *Total* maps are obtained by calculating strains between the initial state *t* = 0 and a deformed state *t* = *n*, thus showing the accumulated strain since the beginning of the triaxial loading test. The difference between incremental and total strain maps is shown in Fig. 10 ▸.

#### 4.4.2. Normalized incremental strain in reference configuration

We started with the observation that both total and incremental maps are needed to understand the full deformation history. Incremental and total strain maps provide complementary insights for local analysis. Total strain maps have the advantage of following the cumulative strain history. Incremental strain maps reveal mechanisms at a specific loading event.

**Figure 11:**
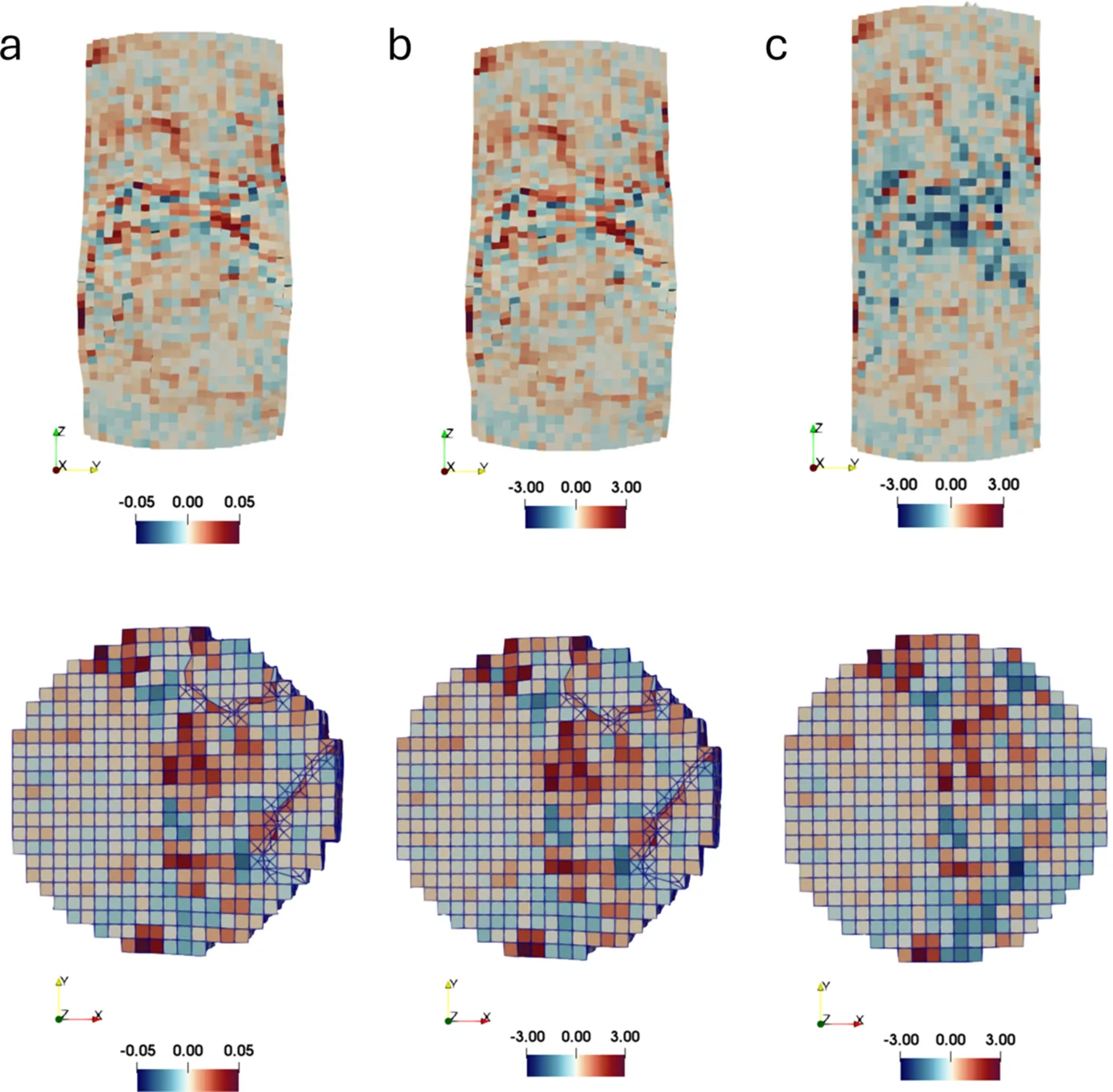
Vertical and horizontal cross-sections of (*a*) incremental volumetric strain maps, (*b*) with normalized local strain and (*c*) put back into the reference configuration.

However, with each increment the reference configuration changes, as seen in Figs. 10 ▸(*b*) and Fig. 11 ▸(*a*). To circumvent this problem, each mesh is put back into the initial reference configuration. In addition, we normalize each SV strain tensor with the global axial strain increment. DVC calculations remain unchanged and these modifications are performed directly on the data files. The whole process is illustrated in Fig. 11 ▸.

## 5. Example of data analysis

**Figure 12:**
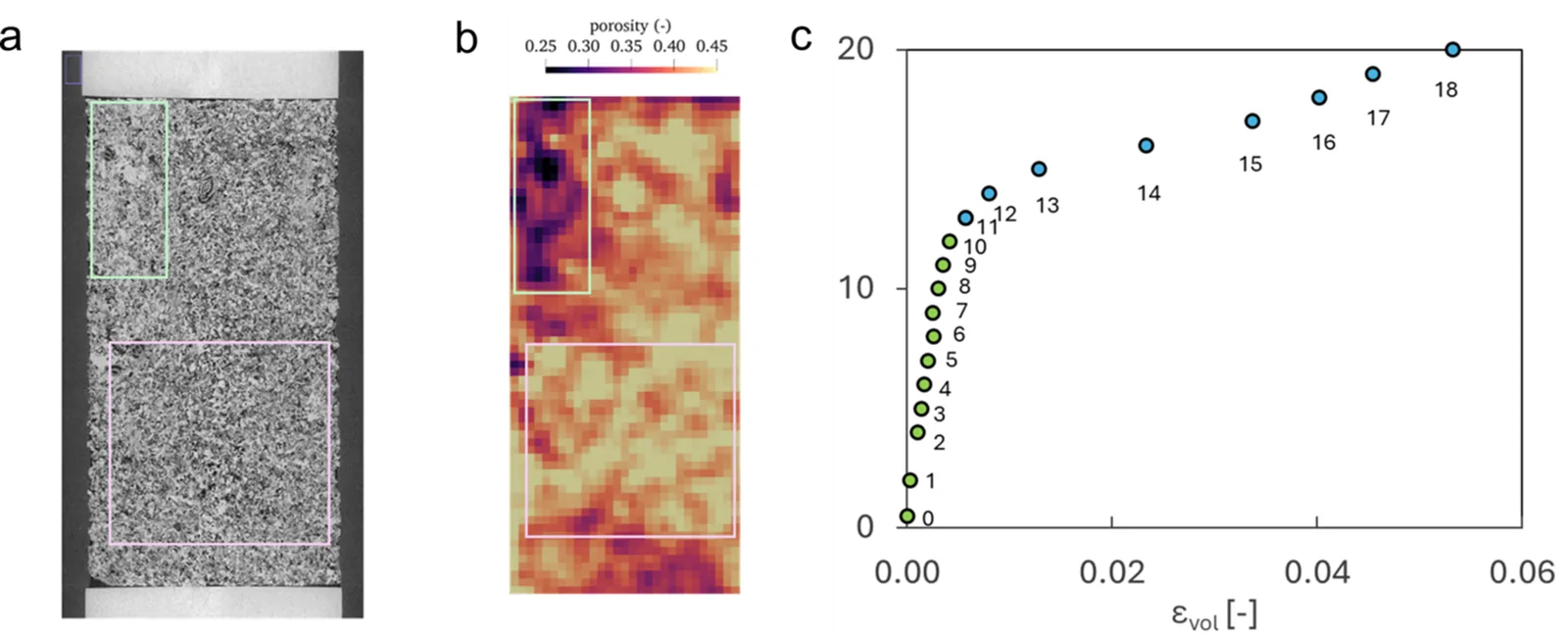
Vertical section of (*a*) the XRCT scan of the sample tested at 20 MPa of confining pressure in undisturbed conditions, (*b*) respective porosity map. (*c*) Mean stress *p* versus volumetric strain ɛ_vol_ for the porous sample tested up to 20 MPa of confining pressure during the hydro­static loading phase.

We apply the method described here to a porous SML sample (42% of initial porosity) tested during the *in situ* campaign at SOLEIL. The sample was loaded in hydro­static conditions up to 20 MPa of confining pressure. The sample was imaged at the initial state. In Figs. 12 ▸(*a*) and 12(*b*) we show vertical slices of the initial microstructure and the corresponding initial porosity map, respectively. The sample is globally homogeneous but presents some denser millimetre-sized zones. Occasional micrometric dense inclusions and large pores are also encountered in the microstructure. A low porosity zone appears in the top-left of the slice [top light green rectangles in Figs. 12 ▸(*a*) and 12(*b*)]. A porous zone [lower light pink square in Figs. 12 ▸(*a*) and 12(*b*)] is also visible, increasing average slice porosity in Fig. 12 ▸(*c*).

**Figure 13:**
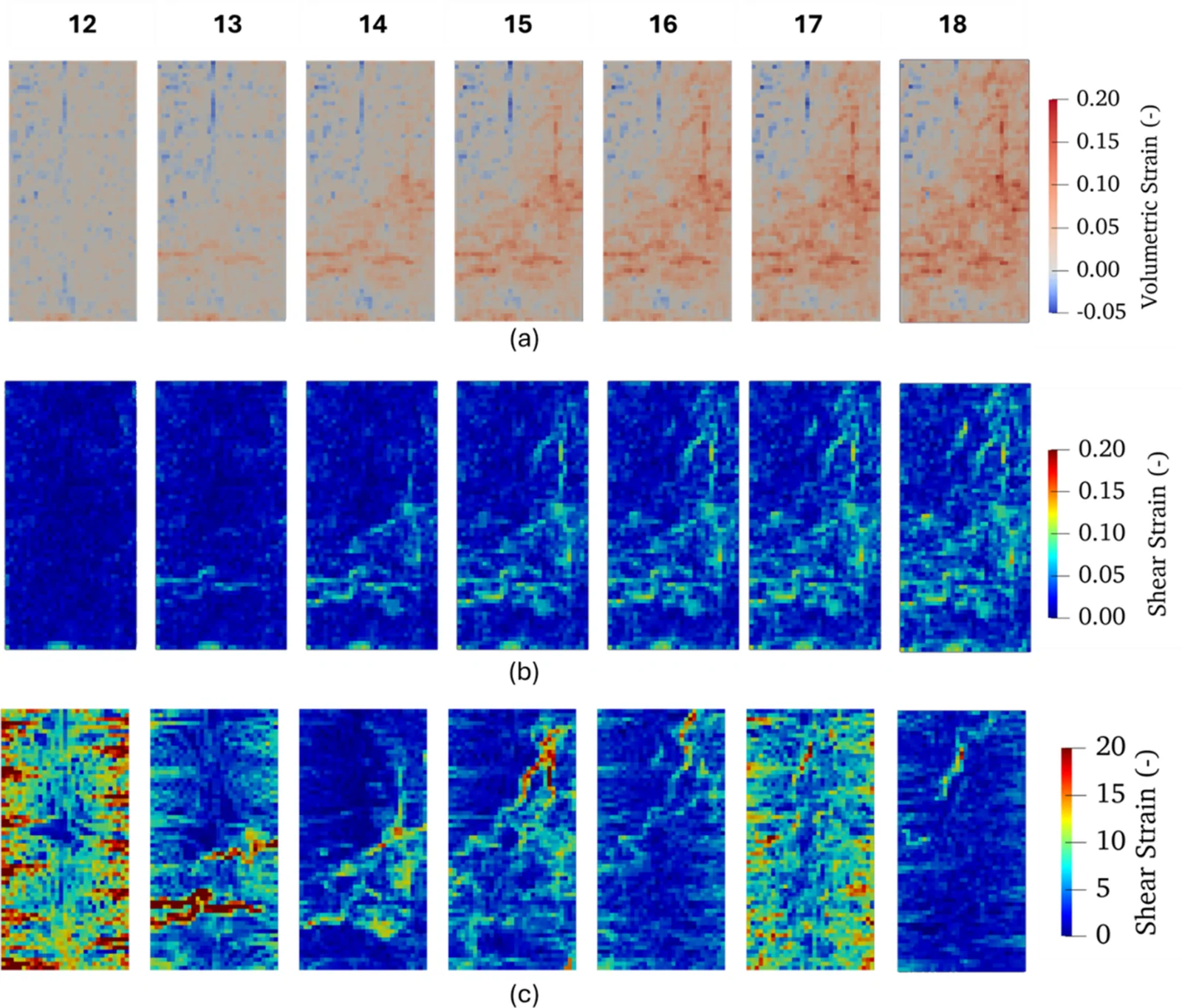
Local strain maps for the porous sample 38a during the hydro­static loading phase. (*a*) Total volumetric strain maps; (*b*) total shear maps; (*c*) normalized incremental shear strain magnitude maps. The numbers of scans on the top of the figures refer to the scans in Fig. 12 ▸(*c*).

Hydro­static loading is applied in 18 steps [points 1 to 18 in Fig. 12 ▸(*c*)]; after each step, the sample is imaged. Fig. 12 ▸(*c*) shows the global mechanical response, with *p* versus volumetric strain ɛ_vol_, obtained with DVC. Initially, the mechanical response is elastic, up to a critical pressure 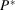 evaluated at 13 MPa (scan 11). Further loading is accompanied by a deviation from linearity, where further increase in confinement is accompanied by stronger compaction. Fig. 13 ▸ shows the total volumetric (*a*) and shear (*b*) strain maps, as well as the normalized incremental shear strain maps (*c*). During the elastic phase, no local compaction was observed. Signs of failure at the bottom of the sample were visible starting step 12 with both shear and compaction. Starting the critical pressure, we observe a diffusion of compaction and shear bands. In the incremental maps, most intense shear is visible in the newly deformed zones, while those previously formed see their intensity drop. This pattern development indicates a failure propagation front. The upper left part remains relatively intact. This zone is linked with the zone of lowest initial porosity [Fig. 13 ▸(*b*)]. Comparing the deformation patterns with the initial porosity map [Fig. 11 ▸(*b*)], local shear and volumetric strain propagate in the most porous zone, while the densest zone remains relatively intact at 20 MPa. Initial porosity seems to define the spatial and temporal variability of strain.

We observe higher strain intensities in higher porosity regions, and low intensities in denser regions. This indicates a strong correlation between initial porosity and deformation. Contrary to a sample showing localized failure, the large number of observations, as seen above, reduces the likelihood that a pattern is a random occurrence. This relationship is thus quantified with the example above.

## 6. Quantitative correlation between initial porosity and strain

**Figure 14:**
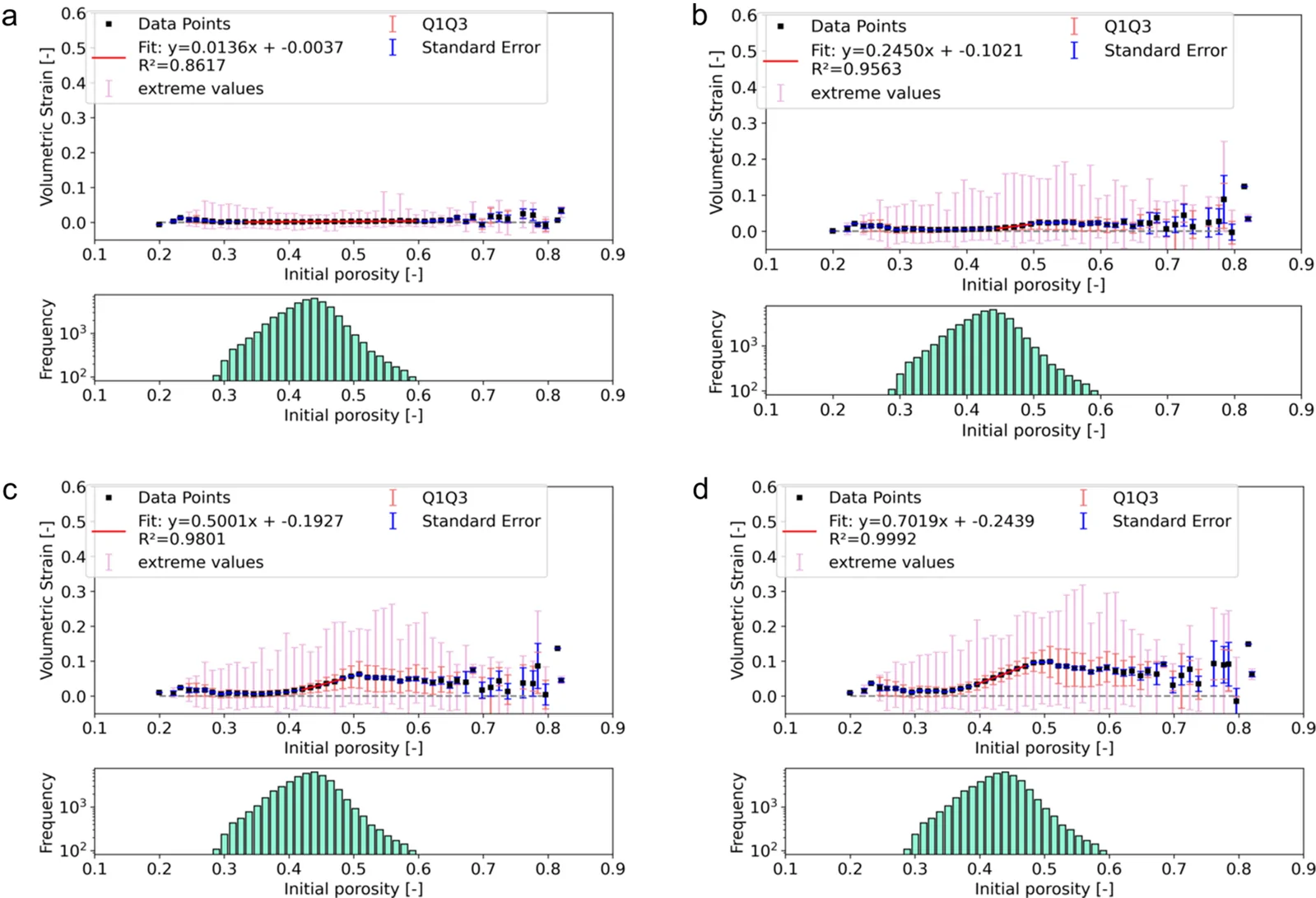
Correlation between initial porosity and volumetric strain for a porous sample tested at 20 MPa of confining pressure: correlation between initial local porosity and volumetric strain for four different steps: (*a*) step 5, at 7 MPa [see Fig. 12 ▸(*c*)]; (*b*) step 12, at 14 MPa (Fig. 13 ▸); (*c*) step 14, at 16 MPa; (*d*) step 18, at 20 MPa. The histogram below shows the initial local porosity distribution, for bins with over 100 data points. The linear fit (red line) correlates initial porosity and volumetric strain.

Initial porosity is assumed to be a key feature in the interpretation of the mechanical behaviour in SML. For each initial position of the reference state, a porosity is associated. Since averaging reduces uncertainties, we grouped local porosity values into 50 bins and calculated statistical descriptors of volumetric strain (namely, the mean, the standard error, and the first and the third quartile) for each bin. With 50 bins, the standard on volumetric strain remains negligible (∼10^−3^), in highly populated bins. However, the average trend is progressively noised out by local effects, which alters the linear fitting. This approach assumes that subvolumes with the same porosity exhibit comparable behaviour in terms of volumetric strain, regardless of their location in the sample. The statistical distribution of the porosity is shown at the bottom of each subfigure in Fig. 14 ▸. The correlation between volumetric strain and initial porosity is presented at the top of each subfigure. The standard error order of magnitude is evaluated at 10^−2^ for bins with under 10^3^ data points. Bins with more than 10^3^ points (accounting for 89% of the total volume) have a 10^−3^ standard error magnitude. The low magnitudes for such bins reinforce the hypothesis that local behaviour is predominantly governed by intrinsic properties rather than local or global boundary conditions. Highest porosities are observed to fail and compact first. With increasing confining pressure, denser porosities fail. We observe that a linear relationship can be fit between initial porosity and volumetric strain for initial porosities ranging between 35 and 50% [Figs. 14 ▸(*b*), 14(*c*) and 14(*d*)], where each bin of this bracket contains a large number of observations (>1000). The quartiles and mean volumetric strain exhibit small differences (around a 10^−2^ order of magnitude, indicating limited skewness and therefore relative behavioural homogeneity). The linearity likely reflects an intrinsic material property in this porosity subset.

## 7. Conclusions

In this paper we presented analysis of 4D X-ray computed tomography data acquired during *in situ* experimental campaign led at SOLEIL. An in-house-developed triaxial press was used for the first time in an *in situ* setting. The press, although having low stress limits, was suitable for exploring all mechanical regimes of our porous carbonate rock. Volumes were analysed with DVC. Error estimation of strains showed a systematic error in volumetric maps and shear strain magnitude maps, resulting from the helical acquisition configuration. Instead of image segmentation, we used mean grey-level-based porosity maps. While our material is not a perfect two-phased material, the estimations remained acceptable, especially in the presence of a microporous phase coming from the initial matrix or grain crushing. This method can be implemented in existing DVC codes to analyse large volumes of data. Additionally, since averaging strain and porosity lead to lower error magnitudes, we were able to show a relevant linear relationship between initial porosity and volumetric strain in some cases.

## Data Availability

The strain and porosity data used for each sample tested *in situ* at the PSICHE beamline (Synchrotron SOLEIL, Palaiseau) presented in the study are available at Zenodo via (Doré-Ossipyan & Quacquarelli, 2025*a* ▸, 2025*b* ▸, 2025*c* ▸, 2025*d* ▸, 2025*e* ▸). All files can be opened and analysed with the open access software *Paraview* (Ahrens *et al.*, 2005 ▸).
